# High-resolution NMR studies of antibiotics in cellular membranes

**DOI:** 10.1038/s41467-018-06314-x

**Published:** 2018-09-27

**Authors:** João Medeiros-Silva, Shehrazade Jekhmane, Alessandra Lucini Paioni, Katarzyna Gawarecka, Marc Baldus, Ewa Swiezewska, Eefjan Breukink, Markus Weingarth

**Affiliations:** 1 NMR Spectroscopy, Bijvoet Center for Biomolecular Research, Department of Chemistry, Faculty of Science, Utrecht University, Padualaan 8, 3584 CH Utrecht, The Netherlands; 2 Institute of Biochemistry and Biophysics, Polish Academy of Sciences, Pawinskiego 5a, 02-106 Warsaw, Poland; 3 Membrane Biochemistry and Biophysics, Bijvoet Center for Biomolecular Research, Department of Chemistry, Faculty of Science, Utrecht University, Padualaan 8, 3584 CH Utrecht, The Netherlands

## Abstract

The alarming rise of antimicrobial resistance requires antibiotics with unexploited mechanisms. Ideal templates could be antibiotics that target the peptidoglycan precursor lipid II, known as the bacterial Achilles heel, at an irreplaceable pyrophosphate group. Such antibiotics would kill multidrug-resistant pathogens at nanomolecular concentrations without causing antimicrobial resistance. However, due to the challenge of studying small membrane-embedded drug–receptor complexes in native conditions, the structural correlates of the pharmaceutically relevant binding modes are unknown. Here, using advanced highly sensitive solid-state NMR setups, we present a high-resolution approach to study lipid II-binding antibiotics directly in cell membranes. On the example of nisin, the preeminent lantibiotic, we show that the native antibiotic-binding mode strongly differs from previously published structures, and we demonstrate that functional hotspots correspond to plastic drug domains that are critical for the cellular adaptability of nisin. Thereby, our approach provides a foundation for an improved understanding of powerful antibiotics.

## Abstract (web summary)

Antibiotics that target the peptidoglycan precursor lipid II are promising templates for next-generation antibiotics. Here authors use solid-state NMR and monitor lipid II-binding antibiotics, such as nisin, directly in cell membranes.

## Introduction

The rapid growth of antimicrobial resistance (AMR) is a severe threat to global health. To date, AMR has been observed against all clinically used antibiotics, which is forecast to cause a staggering 10 million annual human deaths by year 2050^1^. It is hence of pressing need to develop antibiotics that operate via unexploited mechanisms and that are robust to resistance development.

Structural information is decisive for antibiotic design. However, resolving structural information that is relevant for drug development is complicated by the potentially critical influence of the medium on drug-binding modes. Structural data on antibiotic–receptor interactions are therefore ideally obtained under native conditions, which guarantees that the most relevant antibiotic state is studied. This tenet is of special importance for antimicrobial peptides (AMPs) that target bacterial membranes, which are media of enormous complexity. Most of these antibiotics are active in the micromolar concentration range and non-specifically target bacterial membranes. A smaller number of AMPs are active in the nanomolar concentration range, which is achieved by specifically targeting a membrane component such as the essential cell wall precursor lipid II^2^. These lipid II-binding compounds, such as vancomycin, plectasin^3^, tridecaptin^4^, or the recently discovered teixobactin^5^, can kill multidrug-resistant bacteria while resistance development is extraordinarily difficult. Native structural information is of particular relevance for antibiotics that target lipid II, whose accessibility and structure vary across bacteria^6,7^, which can modulate drug activity strongly^3,4,8^. However, structural data on lipid II-binding antibiotics are scarce and usually only available in artificial media such as micelles. Altogether, it is largely unknown how native conditions modulate membrane-binding antibiotics. This is because quantitative structural studies of small (<10 kDa) drug–receptor complexes in cell membranes present major technical challenges that are yet to be overcome.

In principle, solid-state NMR (ssNMR) allows for structural studies directly in cell membranes^9–11^. However, native ssNMR studies are enormously challenging due to the low cellular concentration of the target system and the resulting poor spectral sensitivity. This problem is even more strongly exacerbated for cellular drug-binding studies with lipid II, whose minute native concentration cannot be increased recombinantly. In this work, using a state-of-the-art ssNMR approach that integrates the highly sensitive methods ^1^H-detection and high-field dynamic nuclear polarization (DNP), we show high-resolution studies of an antibiotic–lipid II complex directly in cellular membranes on the example of the lantibiotic nisin^12^.

Nisin employs a unique dual mode of antimicrobial action^12^ and is effective against multidrug-resistant pathogens. Previous solution NMR studies in organic solvents reported a nisin:lipid II complex structure (1WCO)^13^, which served as template for many drug design efforts^14–17^. Here, we show that the native lipid II-bound state of nisin in cell membranes strongly differs from the previously published structure. We rationalize the native conformational space of lipid II-bound nisin and identify plastic domains that enable the antibiotic to adapt to the cellular environment. Intriguingly, these plastic domains correspond to pharmaceutical hotspots that allow to improve nisin’s activity, establishing a link between antimicrobial activity and cellular adaptability. These insights provide a foundation for design strategies for lipid II-targeting antibiotics and demonstrate the high potential of our native structural biology approach to obtain an improved understanding of antibiotics that target membrane constituents.

## Results

### The nisin–lipid II pore only forms in membranes

**Fig. 1:**
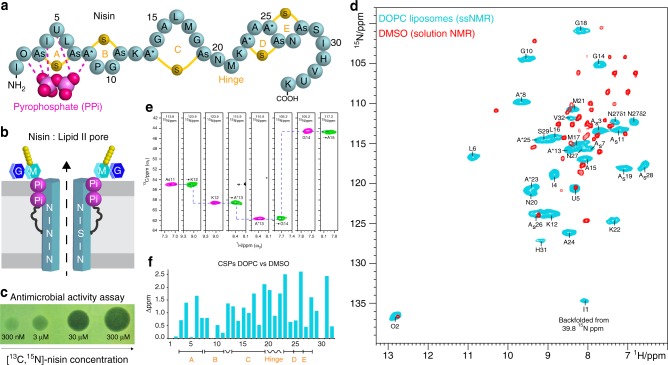
ssNMR experiments of the lipid II–nisin complex in DOPC liposomes. **a** Illustration of the nisin Z peptide–antibiotic. The thio-ether rings are named A–E. Rings A–B are supposed to interact via five hydrogen bonds with lipid II^13^. The non-canonical residues A_s_, A*, O, and U are described in Supplementary Fig. 3. **b** Nisin and lipid II form a defined pore that spans the bacterial plasmamembrane. See Supplementary Fig. 3 for the lipid II structure. **c** A “spot-on-the-lawn” assay shows antimicrobial activity of [^13^C,^15^N]-labeled nisin. **d** Overlay of ^1^H-detected 2D ^15^N–^1^H spectra of the nisin–lipid II complex in DOPC (blue) and DMSO (red). We measured the ssNMR spectrum of nisin bound to lipid II in the pore state (2:1 stoichiometry) in DOPC at 950 MHz (^1^H-frequency) and 60 kHz MAS. The solution NMR spectrum in DMSO was previously published^13^ and shows a 1:1 nisin:lipid II complex. **e** Sequential assignments of lipid II-bound nisin in liposomes. Strip plots are shown from 3D CANH (magenta) and CAcoNH (green) ssNMR experiments. **f** Chemical shift perturbations (CSPs) comparing lipid II-bound nisin in DOPC and DMSO. CSPs were calculated according to ${\mathrm{CSP}}= {\sqrt {\left( \Delta^1H \right)^2+\left( \Delta^{15}N/6.51 \right) ^2}}$

The heavily modified lantibiotic nisin (34 residues) is characterized by five thio-ether rings named A–E (Fig. 1a). Nisin employs a unique antimicrobial dual mode of action that combines pore formation and inhibition of the peptidoglycan biosynthesis analogously to vancomycin^12^. These two functions are structurally separated. Via hydrogen bonds with the backbone amino protons, the N-terminal rings A–B directly bind lipid II at the pyrophosphate (PPi) group, thereby blocking the peptidoglycan synthesis^13^. The PPi group is deemed irreplaceable, and nisin is therefore highly robust against AMR development. The C-terminal part, containing rings D–E, is essential for the subsequent pore formation, in which eight nisin and four lipid II are assumed to span a hole across the plasmamembrane (Fig. 1b)^18^. N- and C-terminal domains are connected via a “hinge” linker, which is common to type A(I) lantibiotics^19^. The hinge is of high pharmacological interest, and mutations/extensions/deletions of hinge residues improve or reduce the pore-forming of nisin^15,16,20,21^. However, in the absence of high-resolution data for the pore, it is unknown how mutations modulate the native state of nisin, critically limiting its use as a template. The only information available is a nisin:lipid II complex solved in DMSO at a 1:1 stoichiometry^13^. Interestingly, the 2:1 stoichiometry, found in lipid membranes^18^, could not be detected in DMSO.

**Fig. 2:**
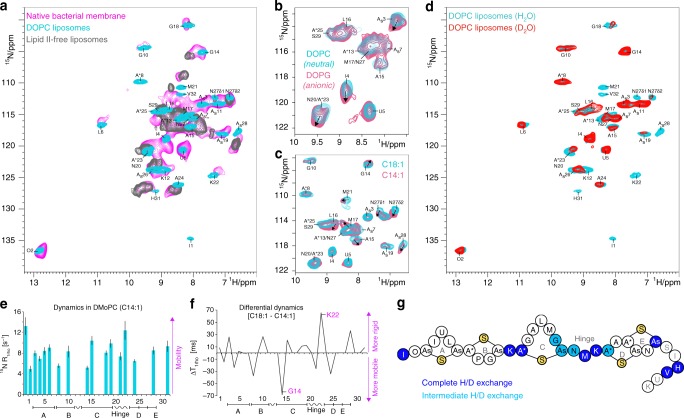
The lipid II–nisin complex in cellular membranes. **a** Comparison of ^1^H-detected 2D ^15^N–^1^H spectra of lipid II-bound nisin in native *M. flavus* membranes (magenta) and in DOPC (cyan). The gray spectrum shows nisin non-specifically bound to DOPG:DOPC liposomes (7:3 ratio) in the absence of lipid II. **b** Overlay of 2D ^15^N–^1^H spectra of lipid II-bound nisin acquired in zwitterionic DOPC (cyan) and anionic DOPG:DOPC (7:3 ratio) (pink) liposomes. **c** Overlay of 2D ^15^N–^1^H spectra of lipid II-bound nisin acquired in C18:1 DOPC (cyan) and C14:1 DMoPC (pink) liposomes. **d** ^1^H/^2^H exchange: 2D ^15^N–^1^H spectra of lipid II-bound nisin acquired in DOPC in fully protonated (cyan) and deuterated (red) buffers. **e** ^15^N R_1rho_ relaxation rates of lipid II-bound nisin acquired in DOPC. The error bars indicate the standard deviation of the fit. **f** Differential ^15^N T_1rho_ times comparing dynamics in C18:1 DOPC and C14:1 DMoPC liposomes. **g** ^1^H/^2^H ssNMR exchange results. Dark and light blue beads represent residues that showed complete and intermediate exchange in deuterated buffers, respectively. Residues S29/I30 and U33/K34 could not be analyzed due to spectral overlap and fast dynamics, respectively

To investigate the structure of nisin bound to lipid II in the pore state, we first produced [^13^C,^15^N]-labeled nisin in the native nisin producer *Lactococcus lactis*. [^13^C,^15^N]-labeled nisin showed strong activity in standard assays (Fig. 1c) and minimum inhibitory concentration (MIC) values comparable to previously reported values (see Supplementary Fig. 4)^22,23^. Afterward, we co-assembled the nisin:lipid II pore at a 2:1 ratio in DOPC liposomes, and acquired a ^1^H-detected 2D ^15^N–^1^H spectrum with 60 kHz magic angle spinning (MAS) (Fig. 1d). We could readily identify the spectrally well-separated signal of residue O_2_, which interacts with the PPi group and exhibits the same very high ^1^H-chemical shift of 12.85 ppm as in DMSO^13^. This demonstrates that nisin is bound to lipid II under our experimental conditions, which is also clearly evidenced by a lipid II-free control sample (Fig. 2a). Lipid II-bound nisin featured sharp, well-resolved NMR signals in DOPC, implying an ordered pore state. Strikingly, the spectra of lipid II-bound nisin in DOPC and in DMSO drastically deviated (Fig. 1d). To analyze this difference, we de novo assigned the backbone chemical shifts of lipid II-bound nisin in liposomes using 3D experiments (Fig. 1e). The chemical shift perturbations (CSPs) profile that compares NMR signals in DOPC and DMSO indeed shows a marked overall conformational change in liposomes. The largest CSPs occur in the C-terminal rings D–E, which is likely diagnostic for pore formation in the DOPC membrane opposed to a non-pore state in DMSO. Moreover, we also observed stark CSPs for rings A–B, which strongly suggests that the critical interaction with the PPi group is altered in liposomes. Intriguingly, in liposomes, we could detect the N-terminal residue I1, which is of high importance for nisin’s activity for uncertain reasons^24,25^, and which remained invisible in DMSO^13^. Altogether, we succeeded to capture the lipid II-bound state of nisin in the pore at high spectral resolution. Our data demonstrate that a previously solved structure^13^ did not report on a physiologically relevant state. However, given that the medium strongly influences the conformation of lipid II-bound nisin (Fig. 1d), we set out to extend our liposomal studies of the nisin:lipid II pore to cellular bacterial membranes.

### The native nisin–lipid II complex in cellular membranes

ssNMR studies in cellular membranes suffer from low molecular concentration, resulting in poor sensitivity. We recently demonstrated that ^1^H-detection can provide the ssNMR sensitivity required to study recombinantly overexpressed proteins in cell membranes at physiological temperatures^26^. However, studying native lipid II–peptide interactions is much more challenging because the lipid II concentration is minute in bacterial membranes (<0.5 mol % compared to plasmamembrane phospholipids)^27^ and cannot be increased recombinantly.

To study the native lipid II-bound state of nisin in the pore by ^1^H-detected ssNMR, we added nisin to membrane vesicles derived from the Gram-positive bacterium *Micrococcus flavus*, a nisin-sensitive bacteria (MIC = 2.7 ± 0.75 nM)^22,23^, which contain all inherent membrane components (lipids, proteins, and other biomolecules)^27^. Our sample contained ~25 μg, i.e., less than 10 nmol of antibiotic. Despite of this 15–20-fold concentration reduction compared to the DOPC sample, we could acquire a sensitive 2D ^15^N–^1^H spectrum of lipid II-bound nisin (Fig. 2a). The native spectrum reproduced our DOPC data, and we could annotate most signals, strongly suggesting that we had captured the native state of lipid II-bound nisin in the pore in both membrane systems. However, subtle differences, which we discuss below, between the spectra of the two membrane systems indicated that the cellular environment modulates the pore conformation. We also detected a second set of signals, which can be seen by the signals splitting for G14 and A_s_28. As it became clear from a negative control spectrum in DOPG/DOPC without lipid II (Fig. 2a, in gray), these signals originate from non-specifically bound nisin, present in slight excess over lipid II, whose exact native concentration is difficult to measure. Note that anionic lipids were used to enhance non-specific binding in the control sample^28^.

Despite the simultaneous presence of lipid II-bound and non-specifically attached nisin, a number of residues (O2, A_s_3, I4, U5, L6, G10, A_s_11, K12, G14, A_s_19, K22 and A_s_28) give unambiguous signals for the native state of lipid II-bound nisin. Interestingly, the amino group of residue I1 was not detectable. This suggests that the I1 amino group has a high mobility, given that we used dipolar ssNMR experiments, in which signal sensitivity decreases with increased dynamics. Generally, rings A–B (O2–G10) showed marginal CSPs in cell membranes compared to DOPC, which is in agreement with their tight interaction with the PPi group, rendering these rings less susceptible to the medium. Exceptions were residues L6 and especially I4, which both showed ^15^N CSPs between 0.6 and 1.0 ppm. Furthermore, the ^1^H-signals of residues L6, G10, and A*8 were broadened, potentially due to heterogeneous PPi interactions. Ring C (A*13–A_s_19) showed clear CSPs in cellular membranes, which are most notable for G14 and A_s_19. Further CSPs for L16–M17 are consistent with the native spectrum but could not be unambiguously resolved. This suggests that the cellular membrane subtly changes the C ring conformation, which is essential for nisin activity for unexplained structural reasons^29^.

Intriguingly, the hinge (N20–K22) and C-terminal domain (A*23–A_s_28), which form the actual transmembrane (TM) pore, featured consistent signal shifts in the cellular membrane compared to DOPC. The hinge is the only TM element that is not restraint in thio-rings and is assumed to play a special role^30^. Drug development efforts have concentrated on the hinge, yielding nisin derivatives with enhanced bioactivity^14–16^. The hinge residues and A*23–A24 in direct proximity, showed clear CSPs in cellular membranes. Intriguingly, M21 either strongly shifted or disappeared in the cellular spectrum due to increased mobility. Altogether, this suggests that the hinge is plastic and important for the adaption of nisin:lipid II pore to bacterial membranes. Furthermore, also the pore-forming rings D–E are sensitive to the membrane environment, as shown by the marked CSP of residue A_s_28. Residues H31–V32 disappeared in the dipolar-based cellular spectrum, pointing to an increased mobility of the C terminus.

### Adaptation of the pore structure to native environment

The differences between the spectra from DOPC or *M. flavus* membranes indicate that the nisin pore structure adapts to the membrane environment. A likely source of this modulation is the complex composition of lipid tails and headgroups in cellular *M. flavus* membranes. In the following, we investigated the impact of the membrane composition on the conformation and dynamics of lipid II-bound nisin, focusing on two key aspects, i.e., bilayer charge and thickness. Furthermore, we varied the length of the prenyl-chain of lipid II.

Gram-positive membranes generally are highly enriched in anionic lipids that constitute usually >50% of the bilayer^31^. To test the effect of an anionic bilayer, we acquired ssNMR data of the lipid II-bound state of nisin in the pore in mixed liposomes composed of anionic DOPG and zwitterionic DOPC lipids (Fig. 2b). Globally, the ssNMR spectrum shows that nisin is only weakly modulated by the membrane charge, in agreement with its high specificity for lipid II^20^. Nonetheless, we observed smaller but clear ^15^N CSPs around 0.5–1 ppm for residues A_s_3–I4, which correspond in direction and magnitude to the signal perturbations in *M. flavus* membranes. This means that ring A at the membrane–water interface is modulated by anionic lipids in cell membranes. Interestingly, this observation agrees with mutagenesis studies that showed that replacing I4 by a cationic residue favorably affects nisin’s activity^32^. Equally interesting, also residues N20 and A*23–A24 at and around the hinge showed significant CSPs in anionic membranes. These residues all showed significant CSPs in *M. flavus* vesicles. Given that the hinge is most likely located in the membrane core and not directly interacting with lipid headgroups^33^, these findings suggest that the hinge conformations change in order to adapt the pore structure to the membrane charge.

Membrane thickness has been shown to be very important for the pore-forming activity of some lantibiotics^34,35^. *Micrococcal* membranes contain mainly branched C15:0 fatty acids and are therefore thinner compared to a DOPC bilayer. To explore the modulatory influence of the bilayer thickness, we acquired spectra of the lipid II-bound state of nisin in the pore in liposomes formed of C14:1 DMoPC that is much shorter compared to DOPC (18:1) (Fig. 2c). While rings A–B, that are not in contact with the lipid tails, did not show CSPs in DMoPC, almost all residues of ring C (e.g., G14 and A15) showed CSPs in the thinner membrane, and these CSPs are consistent with the cellular *M. flavus* spectrum (Fig. 2a). Equally in agreement with the cellular spectrum, residues N27–A_s_28 of the TM part also exhibited clear CSPs in DMoPC. To better rationalize these signal changes, we acquired ^15^N T_1rho_ ssNMR relaxation data in order to compare the mobility of the nisin pore in DOPC and DMoPC liposomes (Fig. 2e, f). From this comparison, we sought to understand if the membrane thickness modulates the conformational dynamics of the pore structure. We observed a striking stiffening of G14 of ring C in DMoPC, which correlated with a stark mobility increase of K22 in the hinge. This corroborates our observation that the hinge is plastic, and suggests that the hinge conformation is coupled to the functionally critical^29^ conformation of the adjacent C ring. Moreover, the C-terminal A_s_28 showed enhanced dynamics in DMoPC, which agrees with the reduced thickness of DMoPC membranes and corroborates our conclusion that the nisin C terminus pierces through the membrane surface. The enhanced C terminus mobility also explains the absence of the H31–V32 signals in *M. flavus* vesicles.

The dodecaprenyl-(C55) prenyl-chain of lipid II is highly conserved in bacteria. Since we used a slightly shorter heptaprenyl-(C35) lipid II for ssNMR measurements in DOPC liposomes, the longer length of the C55-prenyl-chain in the native *M. flavus* membrane could potentially modulate our cellular ssNMR spectra. However, such an influence is not likely, given that previous leakage studies strongly suggest that the tail of the prenyl-chain is neither involved in pore formation nor interacts with nisin^23^. Indeed, the nisin:(C35)–lipid II and nisin:(C55)–lipid II complexes give exactly the same 2D NH spectra in DOPC liposomes (Supplementary Figure 2).

### Linker regions enable the cellular adaptability of the pore

So far, our results pointed to an important role of the hinge plasticity, which may be required for the adaption of the pore to the thickness and charge of the target membrane. In a pore structure, this may mean that the hinge would be accessible by the water phase through the pore lumen. Provided the hinge is not involved in strong inter- or intramolecular interactions, which is expected as it needs to stay flexible, the hinge residues should be sensitive to deuterium exchange^26,36,37^. Indeed, after 1d of incubation in deuterated buffers, signal of G18–A24 around the hinge had either disappeared or showed strongly decreased intensity in a 2D ^15^N–^1^H spectrum (Fig. 2d, g). This demonstrates that the hinge has the required conformational flexibility to enable the adaption of the pore structure to the cellular environment, and it also demonstrates that hinge residues line the pore lumen. Surprisingly, we also observed a complete exchange of K12, and most likely A*13, that link rings B–C. This suggests that these residues also line the pore lumen, and that the orientation between rings B and C exhibits some degree of flexibility, matching with the rearrangement of ring C that we observed in *M. flavus* membranes and shorter lipids (Fig. 2a, c). Notably, just like the hinge, residue K12 was identified as pharmaceutical hotspot^38^. Altogether, this shows that drug domains of high functional significance relate to nisin’s plasticity.

Residues O2–G10 did not exchange in deuterated buffers, which is in excellent agreement with the direct interaction of rings A–B with the PPi group, protecting them from ^1^H/^2^H exchange. Surprisingly, the critical residue I1^24^, thought to tightly interact with the PPi group^25^, disappeared in deuterated buffers. This result makes a strong hydrogen bond between I1 and the PPi group unlikely. To further investigate the role of the I1 amino group, we measured the nanosecond dynamics (^15^N T1) of lipid II-bound nisin in DOPC, which indeed confirmed the high flexibility of the I1 amino group (Supplementary Figure 1), and which also explains the absence of the I1 amino group in cellular conditions (Fig. 2a).

### The native nisin–lipid II complex as seen by DNP-ssNMR

**Fig. 3:**
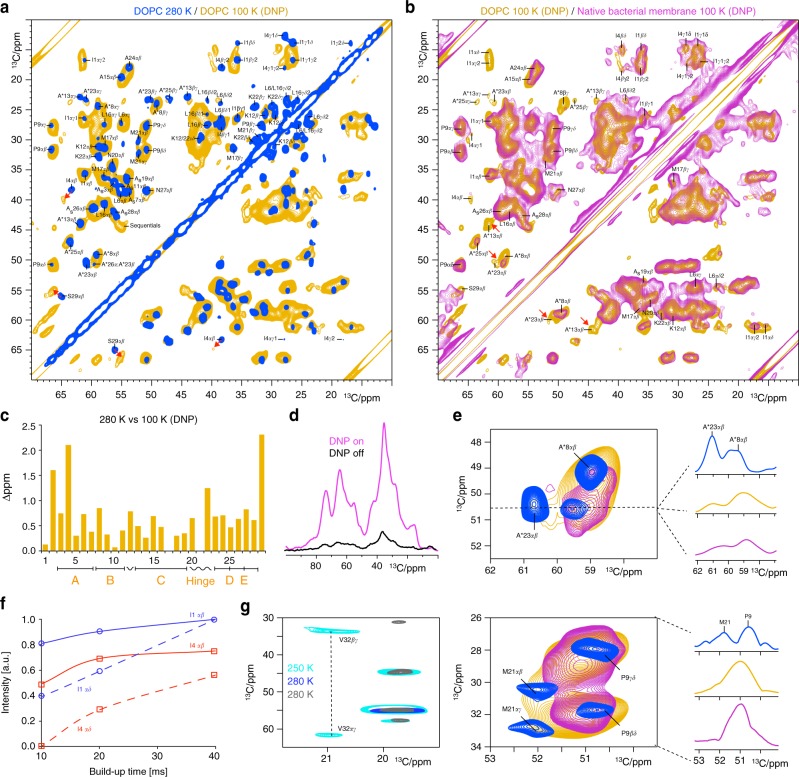
DNP-enhanced ssNMR on the lipid II-bound state of nisin in the pore. **a** 2D ^13^C–^13^C ssNMR spin diffusion spectra of the lipid II-bound state of nisin in the pore in DOPC acquired at 280 K sample temperature (blue) and at 100 K with DNP enhancement (orange). Red arrows highlight strong CSPs. **b** Overlay of DNP-ssNMR 2D ^13^C–^13^C spectra of nisin in the pore acquired in DOPC (orange) and in cellular *M. flavus* vesicles (magenta). Both spectra were acquired at identical conditions using 10.6 kHz MAS, 800 MHz and 40 ms ^13^C–^13^C mixing. **c** Combined (C_α_ + C_β_) CSPs comparing lipid II-bound nisin in DOPC at 280 K against 100 K temperature (DNP conditions). **d** ^13^C cross-polarization spectra of lipid II-bound nisin in *M. flavus* vesicles with (magenta) and without (black) DNP enhancement. **e** The hinge domain is conformationally flexible and broadens out at DNP conditions in DOPC (orange). This is even more pronounced in cellular membranes (magenta). Upper panel: (left) zoom into A*23 adjacent to the hinge; (right) slice through the A*23αβ signal. Lower panel: (left) zoom into M21; (right) projection along the indirect dimension (26–34 ^13^C ppm). **f** ^13^C–^13^C PDSD spin diffusion buildup curves of the C_αβ_ (continuous lines) and C_αδ_ (dashed lines) cross-peaks for residues I1 (in blue) and I4 (red). **g** The mobile nisin C terminus gives faint signals in dipolar 2D ^13^C–^13^C spectra at 280 K (in gray and dark blue with 100 ms and 30 ms ^13^C–^13^C mixing, respectively), while stronger signals appear at 250 K (in cyan, 50 ms mixing)

With the data presented so far, we could capture the native lipid II-bound state of nisin in the pore and rationalize the influence of cellular membranes on nisin’s lipid II-binding mode. However, the ^1^H-detected cellular spectrum did not allow investigating all nisin residues, and did not provide side chain data, which can often be critical for drug binding. In order to obtain this complementary information, we sought to use DNP enhancement, which can boost the NMR signals of biomolecules by orders of magnitude^39–46^. Combined with a high-field 800 MHz magnet^47,48^, we envisioned that DNP would provide sufficient sensitivity and resolution to study the nisin pore in cell membranes. Therefore, we used a similar sample preparation as for cellular ^1^H-detection, where we could clearly detect the native state of nisin in the pore. We obtained a DNP enhancement ε of 8 in *M. flavus* vesicles, which enabled the acquisition of a well-resolved 2D ^13^C–^13^C spectrum of the lipid II-bound state of nisin in the pore in 4 days of measurement time (Fig. 3).

Given that DNP measurements require very low temperatures (100 K), we first explored the impact of cryogenic temperatures in order to subsequently study the influence of cellular membranes on the nisin:lipid II pore. This is because at 100 K, conformational dynamics can result in signal broadening and solvents effects can lead to signal shifts^49–52^. Thereby, DNP spectra can provide valuable information on molecular motions and surface exposure. We first acquired a so-called 2D ^13^C–^13^C “spin diffusion” experiment at 280 K in DOPC to assign the nisin side chain ^13^C-signals (Fig. 3a, in blue). ^13^C-signals of residues I1–V32 could be fully assigned (see Supplementary Fig. 5 and Supplementary Table). Notably, while the C-terminal residues I30–K34 gave only faint signals at 280 K temperature; we could detect these residues at 250 K (Fig. 3g). This confirms the enhanced dynamics of the C terminus that we observed in the ^1^H-detected *M. flavus* spectrum, implying that C terminus sticks out of the membrane.

After obtaining the ^13^C-assignments, we acquired a DNP-enhanced 2D ^13^C–^13^C experiment of lipid II-bound nisin in DOPC at 800 MHz and 100 K as reference spectrum (Fig. 3a, in orange), and compared it to the same type of spectrum acquired in cell membranes (Fig. 3b, magenta). The DNP spectrum in DOPC was of appealing resolution, and globally followed the room temperature (280 K) spectrum closely, which enabled us to analyze most signals at DNP conditions. Figure 3c shows the (C_α_ + C_β_) CSPs in DOPC membranes comparing 280 K and cryogenic (100 K) DNP conditions, which enables us to study which side chains are water-exposed or conformationally heterogeneous. While most nisin residues show minor signal shifts, we see clear maxima for O2 and I4, which is similar in the cellular DNP spectrum. These CSPs likely relate to hydration effects, since ring A presumably localizes on top of the membrane^13^. Note that we also verified with further 2D measurements at 280 K that these CSPs relate to the cryogenic temperatures and are not caused by the DNP radical^53^. Surprisingly, while I4 showed CSPs and signal broadening, the I1 side chain featured intense, defined signals in both DOPC and cellular DNP spectra. This suggests that the I1 side chain is rigid and water shielded in the native pore. This assumption could be further corroborated in a series of dipolar-based 2D spectra at 280 K, showing a much faster built-up for I1 side chain signals compared to I4 (Fig. 3f). This suggests that the I1 side chain, rather than the backbone, may be important for nisin’s pore structure. Note that highly conserved^32^ residue P9 showed intense signals without CSPs in DNP spectra in DOPC and *M. flavus* membranes, demonstrating that the ring B conformation is highly defined, which is presumably critical for efficient PPi binding. Intriguingly, the hinge and adjacent residues like A*23 showed strongly decreased intensities due to signal broadening at DNP conditions in DOPC, which was even exacerbated in cellular membranes (Fig. 3e). This again demonstrates the conformational heterogeneity of the hinge, which is in very good agreement with our ^1^H-detected experiments. Notably, the A*13 signals disappeared on both sides of the diagonal in the cellular DNP spectrum, while these signals were visible in DOPC. This broadening most likely relates to an increased conformational plasticity of residues K12–A*13 that connect ring C to the PPi-binding motif, which is in excellent agreement with the absence of these residues in ^1^H/^2^H exchange experiments. This highlights that ring C is modulated by the membrane complexity, as we showed above (Fig. 2c). Furthermore, at 100 K, residue S29 showed severe line broadening in DOPC and cellular membranes, confirming that the C terminus is dynamically disordered in the native pore. This finding, yet again, underscores the importance of flexible regions for nisin’s activity, given that mutations of S29 increase nisin’s activity^54^.

## Discussion

Antimicrobial resistance is a severe problem and the development of antibiotics is of high urgency. Drugs that target lipid II, the bacterial “*Achilles heel*,” are promising templates for next-generation antibiotics that are robust against AMR^2^. However, the native, i.e., pharmaceutically relevant binding modes of these drugs are scarcely understood because the major technical challenges to study small drug–receptor complexes in cell membranes at the atomic level are yet to be overcome. In the present study, we introduced a cutting-edge ssNMR approach that enables comprehensive high-resolution studies of antibiotic–lipid II complexes directly in native bacterial membranes. We demonstrated our approach on the example of the lantibiotic nisin, which uses a unique dual mode of action of targeted pore formation and vancomycin-like blockage of the peptidoglycan synthesis.

A previous solution NMR study in DMSO already provided a nisin:lipid II complex structure^13^, which has been the gold standard for the structural understanding of nisin and similar lantibiotics. The present ssNMR data conclusively showed that the conformation of lipid II-bound nisin is drastically different in membranes, i.e., the physiologically/pharmacologically relevant environment. De novo ssNMR assignments demonstrated that the membrane environment markedly modulates the nisin:lipid II complex structure, which is because nisin forms a pore complex with lipid II in membranes, as opposed to a non-pore state in DMSO (Fig. 1d, f).

**Fig. 4:**
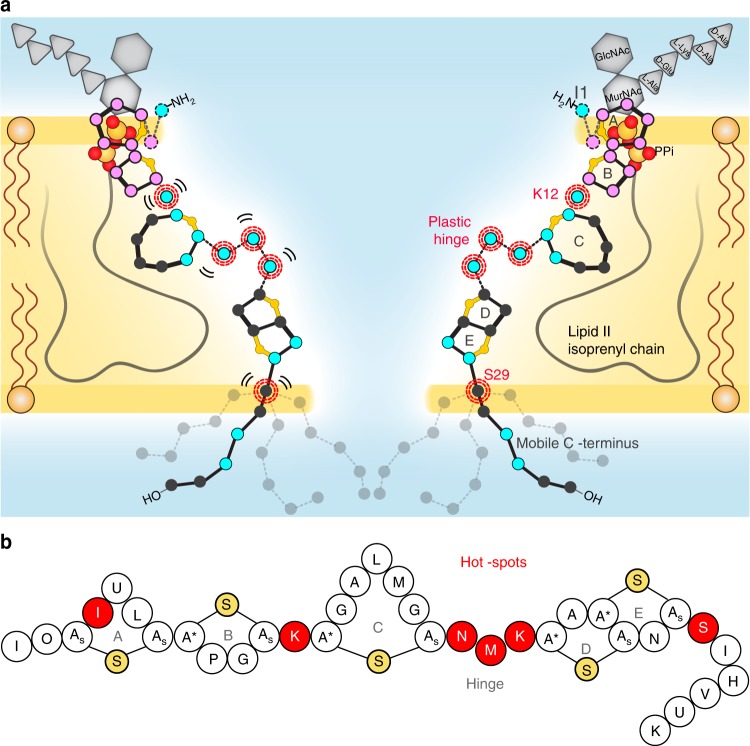
The nisin:lipid II pore topology. **a** Membrane arrangement of the nisin:lipid II topology as seen by ssNMR. Plastic residues that are required to adapt to the bacterial target membrane are highlighted with red circles. Residues that showed ^1^H/^2^H exchange are colored in blue and align the pore lumen. The C terminus is dynamically disordered and resides at the water–membrane interface. The A–B rings (in magenta) interact with the lipid II PPi group. **b** Residues I4^32^, K12^38^, N20–K22^15, 16, 20, 21, 30^, and S29^54^ are pharmaceutical hotspots that enable to improve nisin’s activity upon mutation. These residues were all identified as important for nisin’s cellular adaptability in this study

Given the marked influence of the membrane environment on the binding mode, it seemed plausible that the functional state of the nisin pore is modulated by the cellular membrane complexity. Therefore, as a next step, using ^1^H-detected ssNMR, we could acquire a spectrum of the lipid II-bound state of nisin in the pore directly in native *M. flavus* membranes at physiological temperatures (Fig. 2a). The obtained cellular spectrum globally reproduced our data in DOPC, and demonstrated that we had discovered the functional state of the pore. Intriguingly, we observed that specific regions of lipid II-bound nisin were modulated by cellular conditions (Fig. 4a). Using studies in different membrane types, we showed that the membrane compositions modulate the conformational space of nisin. This modulation is especially visible around the linker K12 and the hinge N20–K22 that connect both lipid II-binding and pore-forming functional motifs of nisin. Using ^1^H/^2^H exchange experiments, we showed that these linker domains are not involved in strong intermolecular interactions, which is required for the plasticity of the linkers (Fig. 2d, e). These observations could be confirmed in high-field DNP-enhanced experiments in native bacterial membranes, where signals of linker domains and the C terminus exhibited severe signal broadening due to conformational heterogeneity. Intriguingly, the linker domains and the C terminus were identified as pharmacological hotspots^15,32,38,54^, and modifications of these plastic elements can strongly increase nisin’s bioactivity, even against Gram-negative pathogens (Fig. 4b)^14,54^. Thereby, our study suggests that these flexible linker regions are critical for nisin to adapt to the target membrane of a given bacterium. This is in excellent agreement with a recent study that indicated that substitutions/extensions of the linker domains change the bioactivity of nisin in a strain-specific manner^30^. Furthermore, our ^1^H/^2^H exchange experiments demonstrate that hinge residues line the pore lumen, which provides the first high-resolution topological insights in the hitherto elusive pore state (Fig. 4a).

Our studies also conclusively showed that the nisin ring A, and especially the conserved ring B^32^, are least modulated by the environment, which is consistent with nisin’s high specificity for the PPi group. Surprisingly, ^1^H/^2^H exchange studies and measurements of nisin’s dynamics suggest that the critical residue I1 does not engage in strong hydrogen bonding with the PPi group^13,25^. While we cannot rule out that the N-terminal I1 amino group transiently interacts with the PPi group, our data hint at an important role for the I1 side chain, which adopts a well defined and presumably water-shielded conformation in the native nisin pore (Fig. 3). This assumption is supported by mutational studies that showed a twofold activity reduction upon replacing I1 by a tryptophan^55^.

To summarize, our native structural study provides high-resolution insights into the dual activity of the preeminent lantibiotic nisin. Given the uniqueness of nisin’s binding mode, its high activity and robustness against AMR development, the use of nisin as a template could be a promising strategy for the construction of antibiotics. As demonstrated in this work, the combination of advanced highly sensitive ssNMR methods paves the way to obtain comprehensive molecular insights into small antibiotic–receptor complexes in a cellular membrane environment. Such native studies may be particularly important to understand the pharmaceutically relevant states of lipid II-binding drugs with a high exposure to the membrane surface^3,5^.

## Methods

### Nisin production and ssNMR sample preparation

The *Lactococcus lactis* NIZO 22186 strain carrying the *nisZ* gene was used for nisin production. Uniformly labeled [^13^C,^15^N]-nisin Z was obtained using [^13^C,^15^N]-enriched medium^56^. Nisin was extracted from the growth medium using Amberlite XAD-16N (Sigma-Aldrich) resin and purified with RP-HPLC (Discovery C18, L. × I.D. 150 mm × 2.1 mm, 5 μm particle size). The column was equilibrated with [H_2_O 95:5 ACN, 0.05% TFA] and elution was carried out with a linear gradient 50–100% of [ACN 95:5 H_2_O, 0.05% TFA] at RT and 0.75 ml flow rate. The absorbance of the eluent was monitored at 214 nm and the fractions containing nisin were subsequently lyophilized. The presence and purity of nisin in the eluted fractions was confirmed by ESI-MS (Finnigan LCQ Deca XP). See Supplementary Fig. 4 for an HPLC elution profile and an ESI ionization trance. The concentration of nisin was measured using the Micro BCA Protein Assay (Thermo Scientific) and cross-validated by ^1^H-solution NMR using DSS as an internal standard. Accordingly, the specific absorbance for nisin Z at 220 nm was 1.214 mg^−1^ ml mm^−1^ in 0.05% acetic acid solution.

Antimicrobial activity of nisin was monitored by a bioassay against *Staphylococcus simulans 22* (Fig. 1c). Accordingly, *S. simulans* was grown in TSB medium (Sigma-Aldrich) at 37 °C under aerobic conditions. Bacteria were seeded at 1% dilution into soft TSB agar medium (0,6% agar, cooled to 37 °C), plated and dried. Aliquot of 10 μl of each test sample was pipetted onto surface of the seeded agar plate, which was afterward dried and incubated overnight at 37 °C. The test samples containing nisin were prepared in Tris buffer (15 mM Tris-HCl, 25 mM NaCl, pH 7.0).

Lipid II (lysine form) was purified using published protocols^23^, using heptaprenyl-phosphate as the polyisoprenyl substrate, as this guarantees optimal pore formation by nisin in DOPC membranes^23^.

Phospholipids 1,2-dioleoyl-sn-glycero-3-phosphocholine (C18:1, DOPC), 1,2-dimyristoleoyl-sn-glycero-3-phosphocholine (C14:1, DMoPC), and 1,2-dioleoyl-sn-glycero-3-phosphoglycerol (C18:1, DOPG) were purchased from Avanti Polar Lipids, Inc.

Dry lipid films containing the lipid II/phospholipids mixture (4% mol/mol) were hydrated by vortexing with 1.5 ml of a nisin solution (15 mM Tris-HCl, 25 mM NaCl, pH 7.0). The correspondent samples contained exact stoichiometric amounts for a 2:1 nisin–lipid II complex. Subsequently, in order to complete nisin–lipid II pore complex formation, the lipid suspension containing the pore complex was incubated at room temperature for 90 min. Liposomes were collected by centrifugation (20,000 × *g*) and loaded into Bruker 1.3 or 3.2 mm zirconia rotors. Afterward, supernatants were virtually free of nisin, as we tested with antimicrobial activity assays. In total, the samples contained ~0.60 mg and 2.60 mg (150 and 750 nmol) of nisin in the 1.3 and 3.2 mm rotors, respectively. Note that we did not observe chemical shift differences for the nisin–lipid II complex in DOPC liposomes with different concentrations (1, 2, or 4 %) of lipid II.

For ^1^H/^2^H exchange studies, reconstituted liposomes containing the nisin–lipid II pores were resuspended in deuterated buffer (99.8% D_2_O, 25 mM NaCl, 15 mM Tris-HCl, pH 7.0) and incubated for 1 day before ssNMR measurements.

Native membrane vesicles (MVs) preparations were obtained from *Micrococcus flavus* DSM 1790 strain based on the method described previously^23^. Accordingly, the bacteria were grown in TSB medium (6L) up to an OD_600_ of 5 (mid log phase). Cells were harvested and washed with 50 Mm Tris-Cl, pH 8.0, resuspended in the same buffer (30–50 ml per liter of culture), and lysed over eight runs using a cell disruptor (Constant Systems). The remaining intact cells were removed by a low-speed centrifugation of the mixture at 600 × *g* and the clear supernatant was centrifuged at 20,000 × *g* to collect the membranes. The resulting membrane pellet was resuspended in the same buffer, flash-frozen in liquid nitrogen, and stored at −20 °C.

During the MVs preparation, a large fraction of the lipid II pool is naturally consumed by the active enzymes present in the membrane. Therefore, the lipid II content in the *M. flavus* MVs was restored to maximal natural amounts by incubating the membranes in a suspension with the corresponding lipid II precursors UDP-MurNAc-pentapeptide and UDP-GlcNAc^12^. The lipid II concentration in the *M. flavus* membrane preparations was estimated to be 0.5% of its total phospholipids molar content^27^, which was calculated via an inorganic phosphate determination. Briefly, membrane lipids were extracted and isolated according to the Bligh–Dyer procedure^57^ and the amount of organic phosphate present was subsequently determined according to the method described by Rouser et al.^58^. Nisin was accordingly added to the lipid II-replenished MVs considering a 2:1 nisin:lipid II stoichiometry.

For the cellular ^1^H-detected ssNMR experiments, *M. flavus* MVs samples contained ~10 nmol of nisin which were loaded in a Bruker 1.3 mm zirconia rotor. For the cellular DNP-ssNMR experiments, *M. flavus* MVs samples contained ~55 nmol of nisin which were loaded in a Bruker 3.2 mm sapphire rotor.

Reconstituted lipid II-bound nisin in DOPC liposomes (4% lipid II) for the DNP samples were prepared using 300 nmol lipid II and 600 nmol of nisin.

Prior to the measurements, all DNP samples were suspended in 60% glycerol-d_8_, 35% buffer solution (25 mM NaCl, 15 mM Tris-HCl pH 7.0 final concentration), and 5% 15 mM AMUPol^53^ (final concentration) in D_2_O. Samples were filled in a Bruker 3.2 mm sapphire rotor.

### NMR spectroscopy

^1^H-detected ssNMR experiments were performed at 60 kHz MAS frequency at static magnetic fields of 18.8 and 22.2 T (800 and 950 MHz ^1^H-frequency, Bruker Biospin). All experiments were performed using dipolar-based sequences at a sample temperature of about 310 K. Sequential backbone chemical shift assignments were performed using 3D CANH, 3D CONH, and 3D CAcoNH experiments^26^. For the latter experiment, one-bond polarization ^13^C–^13^C transfer between CA and CO was achieved with DREAM^59^ recoupling. For all cross-polarization (CP) steps, we used ramped (10–20%) contact pulses. All 3D experiments were acquired with sparse sampling (40–50%). In all ^1^H-detected experiments, the last transfer step from ^15^N to ^1^H was kept short (500–700 μs) to exclusively obtain intra-residual transfer. PISSARRO^60^ low-power (15 kHz decoupling amplitude, 70 μs pulse length) decoupling was used during all indirect and direct detection periods.

Two-dimensional ^13^C–^13^C spin diffusion experiments at room temperature (280 K) were performed with PARIS^61^ recoupling at 950 MHz and 17 kHz MAS frequency. The PARIS recoupling amplitude was 10 kHz, the mixing time 45 ms, and we used the standard phase inversion time of half a rotor period (*N* = ½, i.e., 29.41 μs). SPINAL64^62^ was used in both indirect and direct detection periods. For the dipolar magnetization transfer buildup curves (Fig. 3f), we used 2D PDSD ^13^C–^13^C experiments, i.e., we did not apply a recoupling sequence on the protons in order to emphasize the effect of local molecular motions.

DNP-enhanced 2D ^13^C–^13^C spin diffusion ssNMR experiments were carried out using an 800 MHz/527 GHz setup (Bruker Biospin) at 100 K experimental temperature and 10.6 kHz MAS. A ^13^C–^13^C mixing time of 40 ms was used for all DNP experiments without recoupling irradiation on the proton channel. SPINAL64 decoupling was used in all detection periods.

^1^H-detected ^15^N T_1rho_ and T_1_ relaxation experiments were carried out at 950 MHz and 800 MHz magnetic field, respectively, using 60 kHz MAS^26^. The ^15^N transverse magnetization decay was probed with a ^15^N spinlock field of 17.5 kHz, without application of ^1^H-decoupling during the ^15^N spinlock. We measured six points for both T_1_ (0, 2, 4, 6, 8, 12 and 18 s) and T_1rho_ (0, 10, 25, 50, 100 and 130 ms). Only well-isolated peaks were considered for the analysis, for which we measured the peak intensities. The T_1_ and T_1rho_ trajectories were fit to single exponentials.

## Electronic supplementary material

Supplementary Information

## Data Availability

Data supporting the findings of this manuscript are available from the corresponding authors on reasonable request. The solid-state NMR assignments of lipid II-bound nisin have been deposited in the BMRB (accession number 27572).
