# Supplementary material for: High-resolution NMR studies of antibiotics in cellular membranes
Source: Nat Commun. 2018 Sep 27;9:3963. doi: 10.1038/s41467-018-06314-x (PMC6160437; doi:10.1038/s41467-018-06314-x)
Supplement: Supplementary file 1 — Supplementary Information [file 41467_2018_6314_MOESM1_ESM.pdf]

Supplementary Information

High-resolution NMR studies of antibiotics in cellular membranes

Medeiros-Silva et al.

Supplementary Figure 1

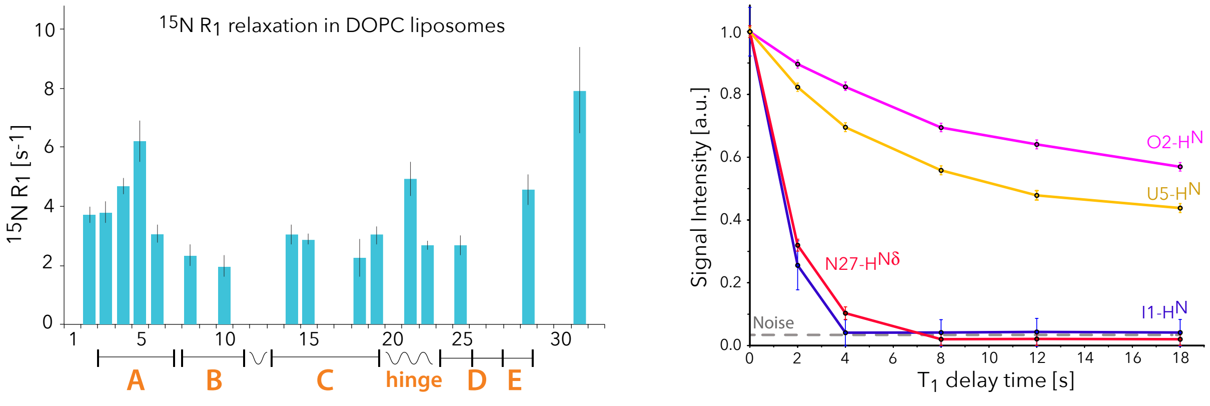

^{15}N R_1 relaxation measurements in Lipid II-bound nisin in DOPC. *(left)* ^{15}N R_1 relaxation rates of Lipid-II bound nisin acquired in DOPC liposomes. The error bars indicate the standard deviation of the fit. *(right)* The N-terminal I1 amino-group (in blue) relaxed very fast, comparable to the N27 side chain (red). Such a fast relaxation is typical for highly flexible side chains that are not, or only weakly, involved in hydrogen bonding. Note that I1 relaxed so fast that we could not fit it, and hence not derive a ^{15}N R_1 rate. The T_1 trajectories were fit to single exponentials according to $I = I^0 + e^{\frac{t}{T_1}}$.

Supplementary Figure 2

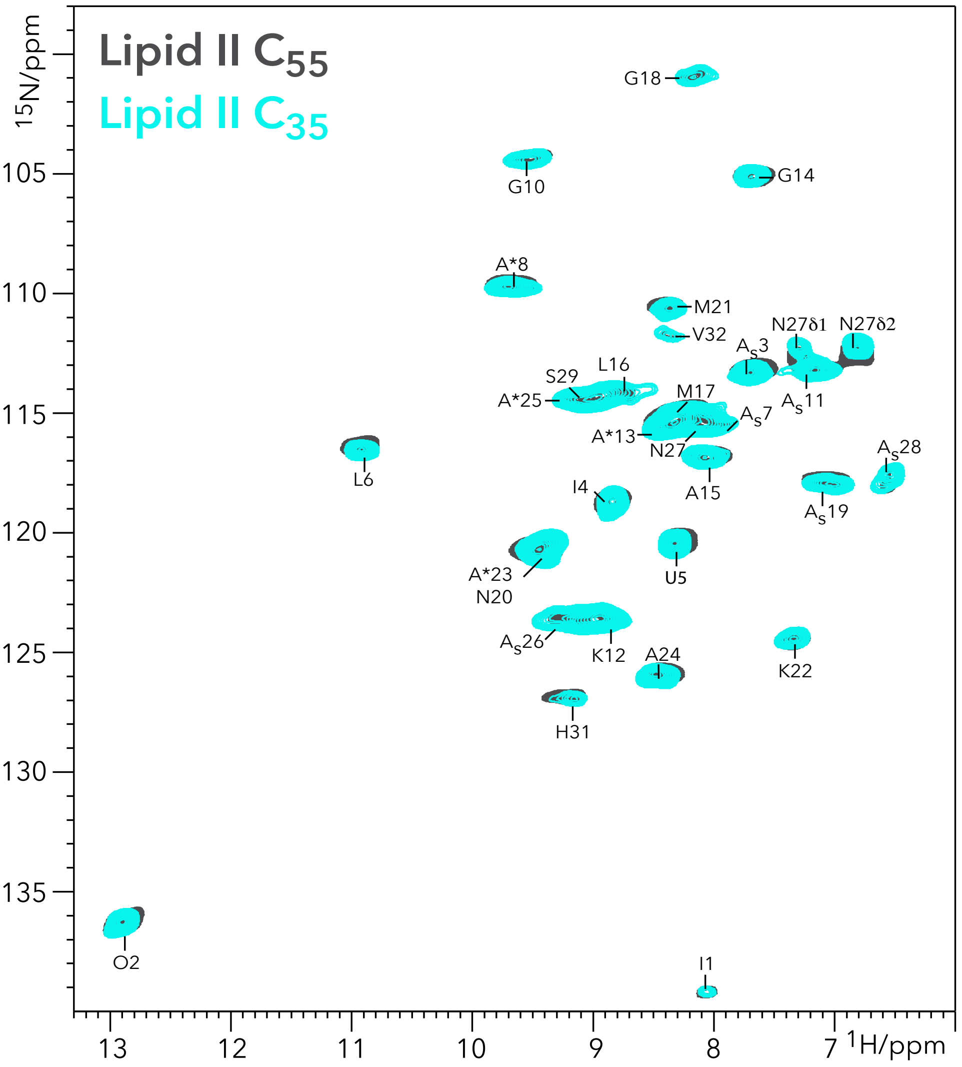

Evaluation of the influence of the length of the Lipid II prenyl-chain on Lipid II-bound nisin. In the cyan 2D NH ssNMR spectrum, we used heptaprenyl-(C35) Lipid II, and dodecaprenyl-(C55) Lipid II in the black spectrum. The spectra, both acquired in DOPC liposomes, show no difference, which demonstrates that the tail of the Lipid II prenyl-chain does not strongly interact with nisin.

Supplementary Figure 3

The non-canonical residues of nisin and a detailed structure of the Lipid II used for this study:

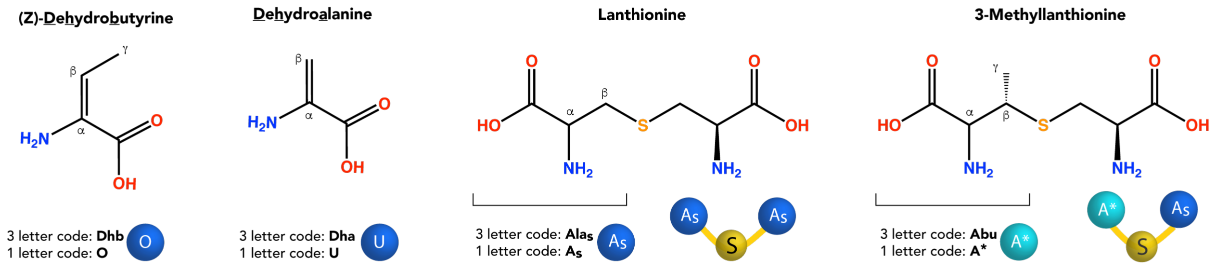

Chemical structures and conventions in this manuscript for the non-canonical amino acids present in nisin.

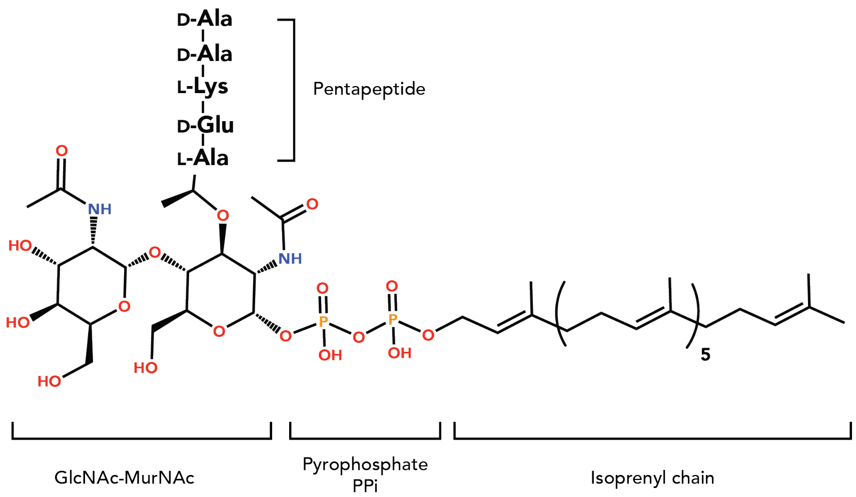

Chemical structure of the Lipid II used for this study.

Supplementary Figure 4

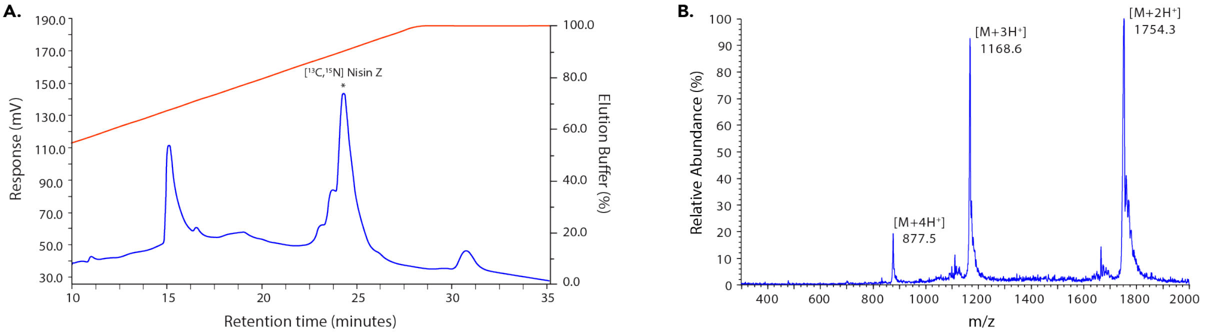

(left) C18 RP-HPLC elution profile of the *L. lactis* growth medium extract. Fractions collected between 24-25 min displayed antimicrobial activity against *S. simulans*. (**right)** ESI ionization trace of the eluted fraction annotated in A.

The purified nisin displayed a MIC of 3.4 ± 0.75 nM (12.0 ± 2.5 μg/L) against *Micrococcus flavus*, which matched that of unlabeled nisin with 2.7 ± 0.75 nM (9.0 ± 2.5 μg/L). These values are consistent with the reported MIC in the literature for *M. flavus*.[1,2]

Supplementary Figure 5

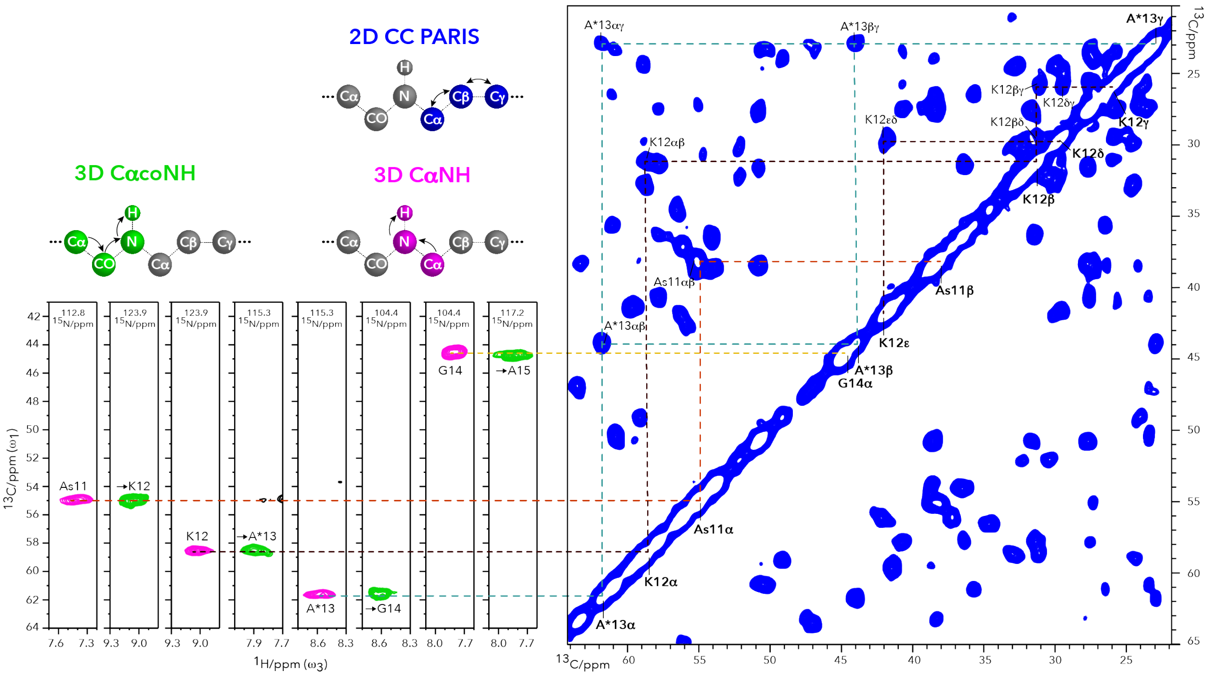

Illustration of the assignment strategy for the 2D ^{13}C-^{13}C PARIS[3] spectrum of Lipid-II bound nisin in DOPC: **(left)** Sequential assignments including Cα chemical shifts were obtained from ^{1}H-detected 3D experiments. **(right)** Thanks to the excellent spectral resolution of the 2D ^{13}C-^{13}C spectrum at 950 MHz magnetic field, knowledge of the Cα chemical shift enabled us to unambiguously assign the complete ^{13}C side chains of almost all residues of nisin. Furthermore, to solve the very few cases where spectra overlap complicated side chains assignments, we also acquired 2D ^{13}C-^{13}C spectra with longer mixing times, as well as a 2D N(C)C experiment. Altogether, we could virtually fully assign the backbone and side chain carbons of residues I1 – V32 of the nisin : Lipid II pore state. Note that we could not observe the two last residues Dha33 and Lys34 at the C-terminus; neither using dipolar- nor scalar-based ssNMR experiments, suggesting intermediate molecular motions.

Supplementary Table 6

Chemical Shift assignments of Lipid II bound-nisin in DOPC liposomes

Table S1: Chemical shift values for nisin Z bound to lipid II in DOPC at pH 7 and approx. 280 K. Values in parts per million (ppm)

Residue	#	N^H	H^N	$H^α$	CO	Cα	Cβ	Cγ	Cδ	Cε
Ile	1	39.8	8.05	4.59	170.6	61.12	35.68	26.56; 16.8	13.83	
Dhb	2	136.6	12.85	- - -	166.9	131.1	134.5	16.03		
D-Ala(s)	3	113.5	7.65	5.8	174.2	56.13	37.2			
Ile	4	119.1	8.83	4.16	174.3	63.3	38.34	27.65; 18.16	12.67	
Dha	5	120.73	8.28	- - -	167.3	134.6	107.8			
Leu	6	116.7	10.85	4-4.3	175.8	55.01	38.68	27.2	26.37; 24.11	
Ala(s)	7	116.5	7.95	5.43	172.9	53.9	38.63			
Abu	8	104.5	9.55	5.05	175.6	59	49.1	24		
Pro	9	138.3	- - -	4.61	177.3	66.61	31.66	27.58	50.75	
Gly	10	104.6	9.49	4-4.5	171.6	44.7				
Ala(s)	11	113.8	7.05	4-4.3	177.5	55.1	38.8			
Lys	12	123.8	9.05	4.4-4.6	177.7	58.73	31.14	26.02	29.56	41.59
Abu	13	115.9	8.32	4.72	174.3	61.87	43.93	22.96		
Gly	14	105.4	7.64	4-4.5	176.15	45.15				
Ala	15	117.3	7.97	4-4.3	180	55.01	19.66			
Leu	16	114.6	8.49	4.2	176.1	57.8	40.76	27.52	25.85; 23.51	
Met	17	115.3	8.25	4.2	176.5	57.8	36.34	31.43		18.2
Gly	18	101.2	8.14	4.12	174.26	44.8				
Ala(s)	19	117.9	7.05	5.2-5.5	173.7	54.06	36.5			
Asn	20	120.8	9.3	4.5-5	173.6	56.59	34.63	177		
Met	21	111.85	8.36	5.2	176.9	52	30	32.3		17.25
Lys	22	124.8	7.33	4.4-4.6	176.6	58.73	32.73	24.53	30.08	42.01
Abu	23	121	9.4	5.11	176.8	60.8	50.5	23.35		
Ala	24	126.1	8.41	3.9	179	53.5	17.97			
Abu	25	114.7	9.05	4.76	176.7	63.5	46.85	23.35		
Ala(s)	26	123.6	9	5.32	173.9	59.7	41.5			
Asn	27	115.6[a]	8	5.25	175.1	50.73	38.5	178.4		
Ala(s)	28	118.2	6.55	4.5-5	173	55.95	42.42			
Ser	29	114.4	8.78	4.5-5	172.8	56.1	64.95			
Ile	30	124.5	9.3	n.d. *	174.2	59.4	38.2	27.78; 17.68	17.68	
His	31	126.4	9.1	n.d. *	174.4	54.72	30.79	129.9**	115.9**	137.5**
Val	32	111.8	8.37	n.d. *	n.d. *	61.60; 60.15	33.3	20.3		
Dha	33	n.d. *	n.d. *		n.d. *	n.d. *	n.d. *			
Lys	34	n.d.*	n.d.*	n.d.*	n.d.*	n.d.*	n.d.*	n.d.*	n.d.*	n.d.*

*n.d. = not defined.

[a] Nδ = 113.3 ppm

**Values obtained via DNP (at 100 K temperatue).

Supplementary References

1 Wiedemann, I. *et al.* Specific binding of nisin to the peptidoglycan precursor lipid II combines pore formation and inhibition of cell wall biosynthesis for potent antibiotic activity. *J Biol Chem* **276**, 1772-1779 (2001).

2 Bonelli, R. R., Schneider, T., Sahl, H. G. & Wiedemann, I. Insights into in vivo activities of lantibiotics from gallidermin and epidermin mode-of-action studies. *Antimicrob Agents Chemother* **50**, 1449-1457, doi:10.1128/AAC.50.4.1449-1457.2006 (2006).

3 Weingarth, M., Demco, D. E., Bodenhausen, G. & Tekely, P. Improved magnetization transfer in solid-state NMR with fast magic angle spinning. *Chem Phys Lett* **469**, 342-348 (2009).
